# Molecular Dynamics Investigation of the Thermo-Mechanical Properties of the Moisture Invaded and Cross-Linked Epoxy System

**DOI:** 10.3390/polym14010103

**Published:** 2021-12-28

**Authors:** Can Sheng, Gai Wu, Xiang Sun, Sheng Liu

**Affiliations:** 1 School of Mechanical Science and Engineering, Huazhong University of Science and Technology, Wuhan 430074, China; cansheng_chongqing@163.com; 2 The Institute of Technological Sciences, Wuhan University, Wuhan 430072, China; wugai1988@whu.edu.cn (G.W.); johnsunx@126.com (X.S.)

**Keywords:** epoxy polymer, moisture, molecular dynamics simulations, thermo-mechanical properties

## Abstract

In spite of a high market share of plastic IC packaging, there are still reliability issues, especially for the effects of moisture. The mechanism between moisture and epoxy polymer is still obscure. A multi-step cross-linking approach was used to mimic the cross-linking process between the DGEBA resin and JEFFAMINE^®^-D230 agent. Based on the molecular dynamics method, the thermo-mechanical properties and microstructure of epoxy polymer were analyzed. In this paper, the degree of cross-linking ranged from 0% to 85.4% and the moisture concentration ranged from 0 wt.% to 12 wt.%. The hydrogen bonds were investigated in the moisture invaded epoxy polymer. Although most of the hydrogen bonds were related to water molecules, the hydrogen bonds between the inside of epoxy polymer were reduced only a little as the concentration of moisture increased. The diffusion coefficient of the water molecules was found to increase with the increase of moisture concentration. When the moisture concentration was larger than 12 wt.% or smaller than 1.6 wt.%, the diffusion coefficient was less affected by the epoxy polymer. In addition, the free volume and the thermal conductivity of the epoxy polymer were considered. It was found that the moisture could increase the thermal conductivity from 0.24 to 0.31 W/m/K, identifying a coupling relationship between moisture and thermal properties. Finally, the mechanical properties of epoxy polymer were analyzed by uniaxial tensile simulation. The COMPASS and DREIDING force fields were used during the uniaxial tensile simulation. A better result was achieved from the DREIDING force field compared with the experiment. The degree of cross-linking was positively correlated with mechanical properties. For the system with the largest degree of cross-linking of 85.4%, the Young’s modulus was 2.134 ± 0.522 GPa and the yield strength was 0.081 ± 0.01 GPa. There were both plasticizing and anti-plasticizing effects when the water molecules entered the epoxy polymer. Both the Young’s moduli and yield strength varied in a large range from 1.38 to 2.344 GPa and from 0.062 to 0.128 GPa, respectively.

## 1. Introduction

As the golden rule facilitating the progress of semiconductor industry, Moore’s Law has guided the industry’s long-term strategy, cost control, and R&D target over the past 50 years. With the semiconductor process nodes decreasing to 7 nm and below, Moore’s Law slows down [1]. However, advanced packaging is probably the answer in the era of “More than Moore”. The new generation electronic products demand high-speed, low-power consumption, and multi-function. These requirements have led to an exponential increase for high-density, high-integrated, and high-reliability packaging. Advanced materials and process should be understood more fundamentally when advance packaging is developed rapidly. Nowadays, plastic packaging has replaced ceramic and metal packaging as the main packaging form. This is because it has the advantages of simpler process, lower cost, and lighter weight. In plastic IC packaging, most of the materials are epoxy polymer [2,3,4,5,6,7,8,9]. Moisture adsorbed by the epoxy polymer can result in delamination, aging, and popcorn problems under high temperature [10,11,12,13,14,15,16]. However, the influence of moisture on the thermal-mechanical properties of epoxy polymer is still obscure. Epoxy polymers with low moisture absorption and high thermal conductivity are expected for harsh service environments. Therefore, it is very important to study the mechanism of the interaction between moisture and epoxy polymer.

Moy et al. [17] conducted an experiment to study the properties of epoxy resin-water interaction. The equilibrium adsorption and diffusion of water in epoxy resin was checked. The experimental results showed that the small moisture concentration strongly concentrates on the specific segments or groups of the polymer. Wong et al. [18] studied the fluidity of water and polymer chains in epoxy materials. Solid-state nuclear magnetic resonance (NMR) technology was used to study the combination of water in the epoxy resin and the plasticizing effect of water. The results indicated that the absorbed water lowers the glass transition temperature of the polymer. The translational mobility of water in epoxy resin was studied by measuring the diffusion coefficient. The water in the rubbery epoxy resin had a higher mobility than the water in the glassy epoxy resin. They pointed out that there were two forms of water molecules in the epoxy resin after moisture absorption. One was free water molecules, which were filled inside the van der Waals volume. It did not cause swelling and deformation of the epoxy resin. The second was the bound water molecules combined with the polymer through hydrogen bonds, which caused the swelling of the plastic encapsulation material. Qiu et al. [19] decorated the tip of the atomic force microscope with terminal CO molecules to provide a higher resolution for the bonding of adsorbed molecules. Non-contact atomic force microscopy (NC-AFM) was used to visualize the hydrogen bonds of 8-hydroxyquinoline (8-hq) molecular components on Cu (111) substrates in real space. A picture of hydrogen bonds was observed for the first time. Starkova et al. [20] quantitatively characterized the water absorption and hydrothermal aging effects on amine cured epoxy adhesives. Hydrothermal aging caused a significant decrease in sample performance. Zhang et al. [21] measured the moisture concentration and diffusion coefficient of an amine cured epoxy polymer by means of gravity measurement. FTIR, DSC, and 2D correlation were used to obtain the formation sequence of hydrogen bond. They concluded that the diffusion coefficient was related to the number and distribution of large-size voids.

According to the experimental method, the hygroscopic process is slow, while the cross-linking reaction process is fast. Hence, it is difficult to observe the specific process in microscopic change and find out the interaction mechanism. Therefore, molecular dynamics simulation can be used as an alternative method to study the microscopic process and analyze the mechanism. The structure and thermal-mechanical properties of polymer using molecular dynamics method have been reported in some literatures. Masoumi et al. [22] studied the cross-linking algorithm for epoxy and curing agents. They analyzed the cross-linking structure using the RDF (Radius Distribution Function). Additionally, the CTE, *T*g, and elastic constants were calculated. Yarovsky and Evans [23] studied the curing process and analyzed the interaction of epoxy polymer with other invading molecules. Sun et al. [24] improved the cross-linking algorithm. Based on the improved cross-linking algorithm, they considered the angle constraint, which could make the structure more reasonable. They obtained *T*g, CTE, and Young’s modulus which were in agreement with experimental values. Srebnik et al. [25] used united-atom molecular dynamics to predict the thermodynamic and mechanical properties of industrial epoxy resins. Their results were in agreement with the experimental measurements. They discussed the fluctuation of the results in detail.

The change of property and its reason in different situations should be analyzed due to the various application environments of epoxy polymer. The influence of moisture on thermal-mechanical properties has become the focus of a few studies. Masoumi et al. [26] analyzed the influence of moisture on the Young’s modulus and thermal properties. The diffusion coefficient was calculated. A tiny amount of moisture contributed to the increase of Young’s modulus. However, when the moisture concentrations became larger, the Young’s modulus decreased. This phenomenon has also been found in other works [27,28,29,30]. Tam [31] analyzed the effect of moisture on the mechanical properties of epoxy/carbon-nanotube nano-composites. They analyzed the mechanism of the interaction between moisture and the epoxy polymer by focusing on the hydrogen bonds. They found that moisture affected the mechanical properties mainly in the way of forming hydrogen bonds with nano-composites when the moisture concentration was low. As the moisture concentration became larger, the hydrogen bonds generally formed between water molecules. Moisture at the epoxy-SWCNT interface affected the intermolecular interaction and damaged the mechanical properties. Guha et al. [32] analyzed the influence of moisture on DGEBA-DETA system with different degrees of cross-linking. Their results showed that as the degree of cross-linking increased, the free volume and polarity also increased. The increase of polarity contributed to the formation of more hydrogen bonds between epoxy polymer and water molecules. The increase of free volume caused the water molecule to aggregate.

The performance requirements for epoxy polymer vary greatly for different applications. Moreover, the thermal-mechanical properties and structure of epoxy vary greatly due to the differences of epoxy matrix, hardener, filler, degree of cross-linking, and moisture concentration. In particular, there is no consistent conclusion about the effect of moisture on epoxy polymer. According to the application requirements of epoxy polymer in the field of electronic packaging, the following simulation was carried out, providing a reference for the selection of electronic packaging materials. In this paper, the structure and thermal-mechanical properties of the moisture-invaded epoxy polymer were studied by molecular dynamics simulation. Firstly, the cross-linking algorithm for epoxy resin and curing agent were discussed. Then, the thermal-mechanical properties and structures of the epoxy polymer were analyzed, including the hydrogen bonds, the free volume, the diffusion coefficient of water molecules, and the thermal conductivity coefficient. Finally, the uniaxial tensile simulations were carried out. The influence of degree of cross-linking and moisture concentration on the mechanical properties of epoxy polymer was analyzed.

## 2. MD Simulation Methodology

### 2.1. Composition of Simulation Cell

**Figure 1:**
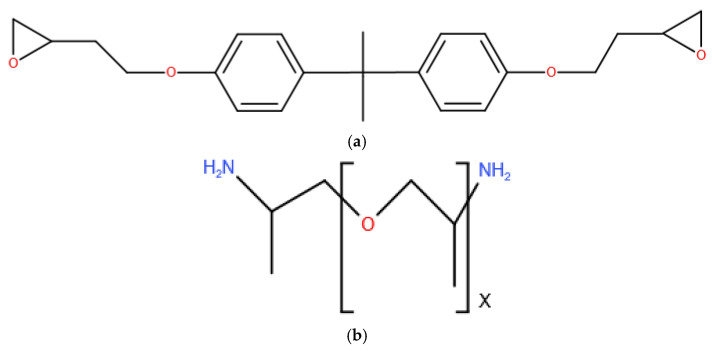
Molecular structure diagram of (**a**) DGEBA and (**b**) JEFFAMINE^®^-D230 (x = 2.6) molecule.

The 48 diglycidyl ether bisphenol-A (DGEBA) molecules and 24 JEFFAMINE^®^-D230 curing agent molecules were used to construct the epoxy polymer. As shown in Figure 1a, one DGEBA molecule possesses two epoxide groups at each end of the monomer. One JEFFAMINE^®^-D230 molecule includes two primary amines, which are shown in Figure 1b. It can cross-link with two DGEBA molecules.

### 2.2. Cross-Linking Algorithm

**Figure 2:**
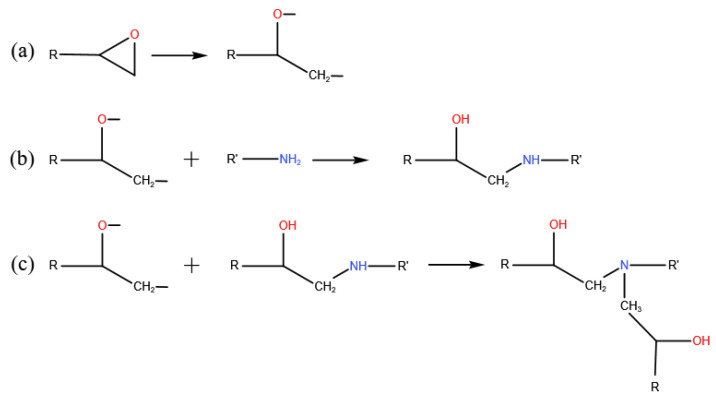
Cross-linking process of DGEBA/JEFFAMINE^®^-D230 system: (**a**) The opening of the epoxide ring. (**b**)The creation of bonds between the epoxy and the primary amine. (**c**) The creation of bonds between the epoxy and the secondary amine.

The curing process is illustrated in Figure 2. In this work, the hydrogen on the primary amine and the secondary amine were assumed to be equivalent. It includes the opening of epoxide ring shown in Figure 2a, and the creation of C-N bonds and the transformation of hydrogen from N to O shown in Figure 2b, c. More than one cross-linking method has been used to simulate the cross-linking process. Most of the methods have been found to produce only a small difference on the results of thermal-mechanical properties [33]. Among them, a multi-step cross-linking approach is widely used [22,26,34]. This approach searches for atoms that can participate in the cross-linking reaction by gradually increasing the cut-off radius. When the cut-off radius increases to the maximum value, it stops building more crosslinking bonds. Then, the system with the largest degree of cross-linking under the current conditions is obtained. During the cross-linking process, the multi-step cross-linking approach firstly seeks for the atomic pairs with the specially labeled C in the epoxide ring and N in the amine group. The process of cross-linking must meet two conditions. One is that the distance between the C and N should be less than the current cut-off radius. The second is that there is at least one H on N. Finally, the cross-linking bond is realized by breaking the C-O bond in the epoxide ring and establishing C-N and O-H bonds.

**Figure 3:**
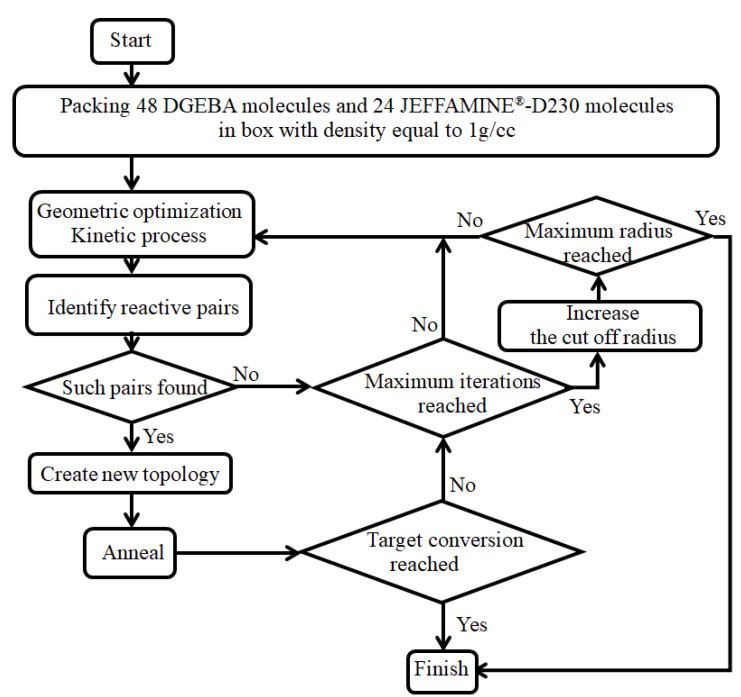
Flowchart of the multi-step cross-linking algorithm.

**Figure 4:**
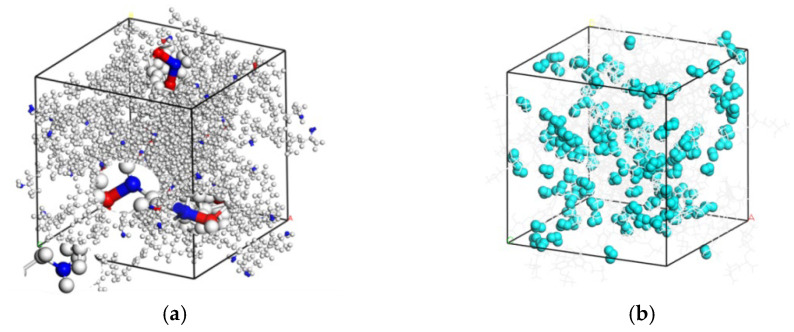
DGEBA/JEFFAMINE^®^-D230 cell with a degree of cross-linking of 85.4% (**a**) without water molecule and (**b**) with moisture concentration of 12 wt.%. All the N are blue. The cross-linked C is red. Some of the cross-linked bonds are magnified. The light blue molecules are water molecules.

In this work, the cut-off radius ranged from 4 Å to 7 Å with a step size of 1 Å. When the cut-off radius met the maximum value of 7 Å, the model with the maximum degree of cross-linking of 85.4% under the current conditions was obatined. As shown in Figure 3, considering the cut-off radius was not the real bond length, the multi-step cross-linking algorithm included a geometric optimization, NVT, and NPT kinetic equilibrium and annealing. Extra pairs of atoms may participate in the cross-linking under the same radius after kinetic equilibrium, which was similar to the experimental process of “stirring”. Since only the distance between the atoms was taken into account in this method, the phenomenon of benzene ring puncture should be checked. In this paper, the structures with degrees of cross-linking of 0%, 38.5%, and 85.4% have been created, and the model with the maximum degree of cross-linking of 85.4% could be shown in Figure 4a. Several kinds of cross-linked bonds were particularly magnified. During the cross-linking process, one N atom may cross-link 0–2 times. Because of the periodic structure, atoms may cross-link with the atoms in the neighbor period. Based on the cross-linked model with a degree of cross-linking of 85.4%, water molecules were randomly added into the cell. Then, eight systems were constructed with moisture concentration ranging from 0 wt.% to 12 wt.%. The model with the maximum moisture concentration of 12 wt.% could be shown in Figure 4b.

### 2.3. Force Field and Simulation Details

To establish the crosslinking system, Amorphous Cell Module of BIOVIA Materials Studio 2017 (MS2017) [35] was used. The COMPASS force field [36] was used in all of the calculations in this article. In addition, the DREIDING force field [37] was also used for uniaxial tensile simulation. The Souza-Martins barostat [38] was used for uniaxial tensile simulation in order to set different stress loads, and the Berendsen barostat [39] with the pressure coupling time constant of 1 ps was used for other simulations. The Nose-Hoover thermostat [40] with a Q ratio of 0.01 was applied to control the temperature. The time step was set as 1 fs in all the dynamic simulations. Periodic boundary conditions were applied in x, y, and z directions. The Smart algorithm was used for geometric optimization [41]. The model was processed with a geometry optimization and a NVT-MD equilibration for 50 ps as well as a NPT-MD equilibration for 2000 ps. For other parameters, the default value was used, which can be found in the manual embedded in MS2017[41].

**Figure 5:**
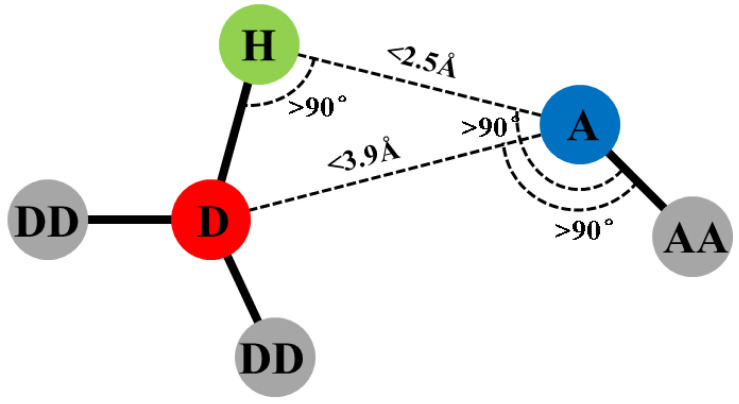
Geometric criteria for hydrogen bonds. D is the donor atom, H is hydrogen and A is the acceptor. DD is the donor antecedent and AA is the acceptor antecedent.

**Figure 6:**
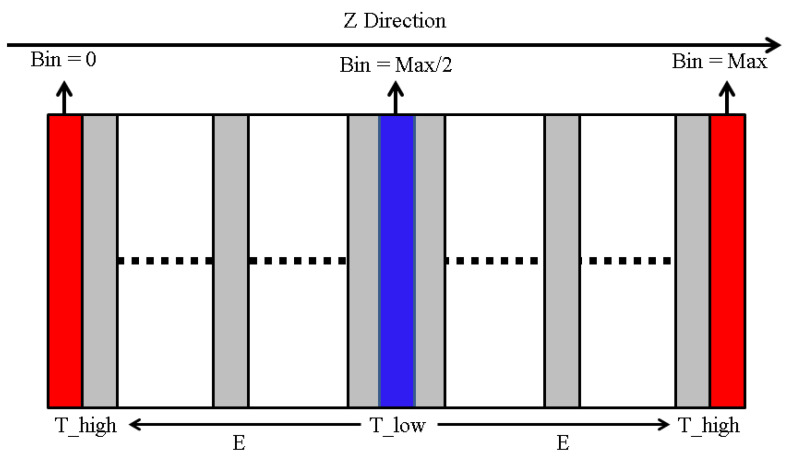
Schematic diagram of the thermal conductivity calculation method.

As shown in Figure 5, according to the geometric rule of hydrogen bond [42], the largest hydrogen bond length was 2.5 Å and the minimum donor-hydrogen-acceptor angle was 90°. The hydrogen bond was obtained through the hydrogen bond calculation function in MS2017 [35]. The free volume of the epoxy polymer was obtained by the Atom Volumes and Surface Tools in MS2017 [35]. The theoretical basis of free volume was the Connolly Surface Algorithm [43]. This algorithm used a sphere with particular radius to probe the surface of the molecules. In this work, the Connolly radius was 1.4 Å and the grid resolution was 2.5 Å. To obtain the thermal conductivity, as shown in Figure 6, the cell was divided into 40 bins along the z direction. Then, 1000 NVT-MD equilibrations and 2000 NVE-MD equilibrations with 250 steps every time were carried out. In the NVE-MD calculation, the velocity of the slowest atom in the middle area and the fastest atom in the side area were exchanged [44]. Then, the energy exchange caused by velocity exchange was obtained. Since our cell was periodic in the z direction, the bin in the middle of cell turned into a cold area and the bins on both sides turned into a hot area. Two temperature gradients from both sides to the middle of the cell along the z direction were formed. Finally, according to the Fourier’s law, the exchanged energy and temperature gradient were used to calculate the thermal conductivity. In uniaxial tensile simulation, NPT-MD equilibration of 100 ps was performed three times for the initial structure. Then, NPT-MD equilibration of 380 ps was carried out under each stress load, which was outputted once every 100 fs. The results of the last 80 ps were used.

## 3. Results and Discussion

### 3.1. Effects of Moisture on the Structure of Epoxy Polymer

In Section 3.1.1, the interaction between water molecules and epoxy polymer was studied by analyzing the hydrogen bonds. Then, in Section 3.1.2, the free volume was analyzed. In Section 3.1.3, the MSD (Mean Square Displacement) of water molecules in epoxy polymer was calculated.

#### 3.1.1. Analysis for the Hydrogen Bond

**Figure 7:**
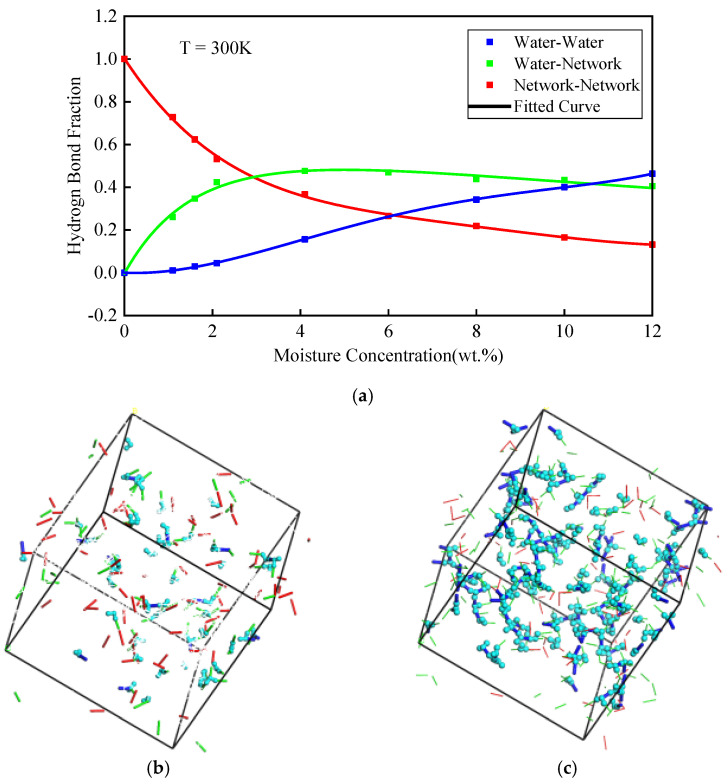
(**a**) Ratio of hydrogen bonds with different pairs of donor and acceptor for various moisture concentrations. Hydrogen bond at moisture concentration of (**b**) 4.1 wt.% and (**c**) 12 wt.%. The red lines are NN hydrogen bonds. The green lines are WN hydrogen bonds. The blue lines are WW hydrogen bonds. The light blue balls are water molecules.

Hydrogen bonds were divided into three types: (1) The Water-Network type of hydrogen bonds, the acceptor and donor of which come from the water and epoxy polymer molecules (WN hydrogen bonds). (2) The Water-Water type of hydrogen bonds, the acceptor and donor of which both come from water molecules (WW hydrogen bonds). (3) The Network-Network type of hydrogen bonds, the acceptor and donor of which both come from the epoxy polymer molecules (NN hydrogen bonds). The number of each type of hydrogen bond was calculated by the average value over the last 1.5 ns, which are listed in Table 1. As shown in Figure 7a, the ratio of various types of hydrogen bonds to the total hydrogen bonds was analyzed. Moreover, the hydrogen bonds were magnified and are shown in Figure 7b,c. As shown in Figure 7a, with the increase of moisture concentration, the ratio of WN hydrogen bonds gradually reached the maximum value. It began to show a decreasing trend at moisture concentration of 6 wt.%. The ratio of WW hydrogen bonds increased linearly, indicating that there existed water clusters. The ratio of NN hydrogen bonds decayed exponentially before moisture concentration of 6 wt.%. These trends were consistent with the results in a previous research, which proved that our structures were reasonable [26]. As shown in Table 1, the number of NN hydrogen bonds did not decrease a lot. Even the moisture concentration reached the maximum value of 12 wt.%, the NN hydrogen bonds only reduced by 38.57%. This showed that the hydrogen bonds between polymers were not easily broken by water molecules, which is consistent with the conclusion in the previous work [45].

#### 3.1.2. Free Volume

Hirschfelder et al. [46] first proposed the concept of free volume in 1937. Through the efforts of Ferry and Flory [47], a relatively perfect free volume theory was formed. The free volume theory refers to the holes between the skeleton inside the material, which could provide space for the movement of molecular chains. The space occupied by molecular chains is the occupied volume. The free volume fraction and free volume size of the amorphous polymer materials may affect the properties, such as the *T*g and the diffusion coefficient [21].

**Figure 8:**
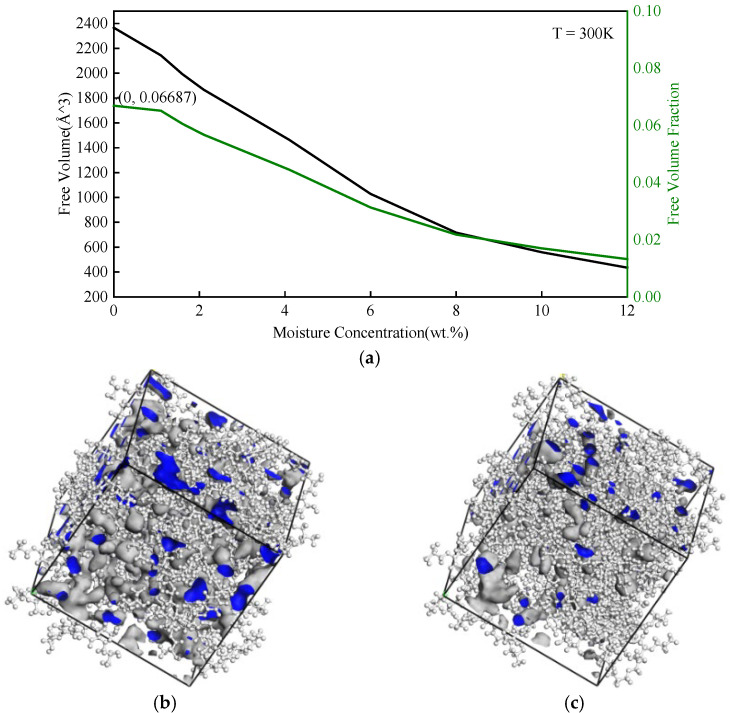
(**a**) Free volume of various moisture concentrations. Free volume distribution at moisture concentration of (**b**) 0 wt.% and (**c**) 12 wt.%. The envelope on the blue side is free volume.

The free volume is depicted in Figure 8, where the black represents free volume and the green lines represent the proportion of free volume to total volume. The free volume decreased as the moisture concentration increased, which means more space was occupied by water molecules. The reduction of the free volume limited the movement space of the polymer segments, which may affect the thermo-mechanical properties of epoxy polymer.

#### 3.1.3. Mean Square Displacement

**Figure 9:**
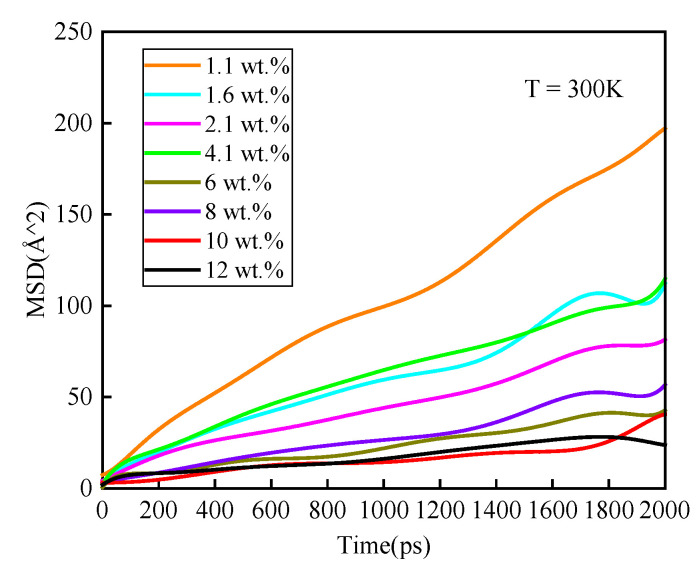
Mean square displacement of water molecules for various moisture concentrations.

**Figure 10:**
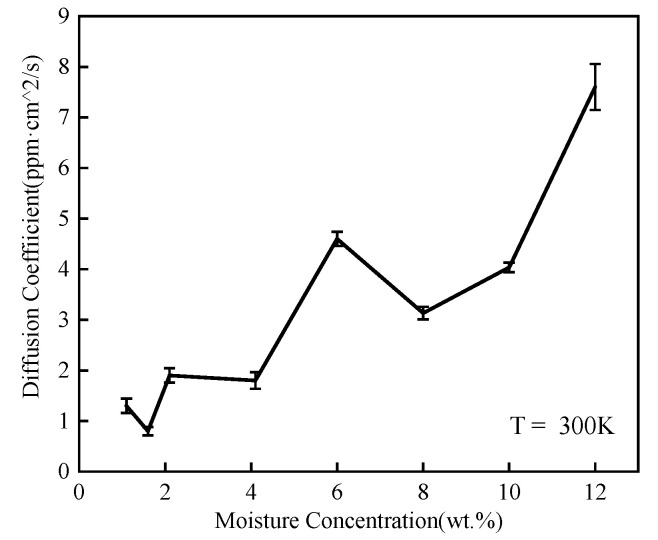
Diffusion coefficients for various moisture concentrations.

In an equilibrium system, molecules can only collide in a small free volume in a short time, but they will jump out of the constraint region and reach another space in a longer time scale. This process can be described by the Einstein relation:

$$\langle r_{i}^{2}\rangle \;=\;6D\cdot t+C$$

where <*r_i_*^2^> denotes MSD, among which, *r_i_* is the position vector of atom *i*. *t* is the sign of time. *D* and *C* are constants, where *D* is the diffusion coefficient. The MSD of water molecules in the epoxy polymer with various moisture concentrations are depicted in Figure 9. As shown in Figure 10, the average diffusion coefficient over three calculations increased in a fluctuated way as moisture concentration increased. When the moisture concentration was above 10 wt.%, the average diffusion coefficient increased exponentially and was more than seven times over the lowest value in this article. It was consistent with that the existence of many WW hydrogen bonds at this moisture concentration.

**Figure 11:**
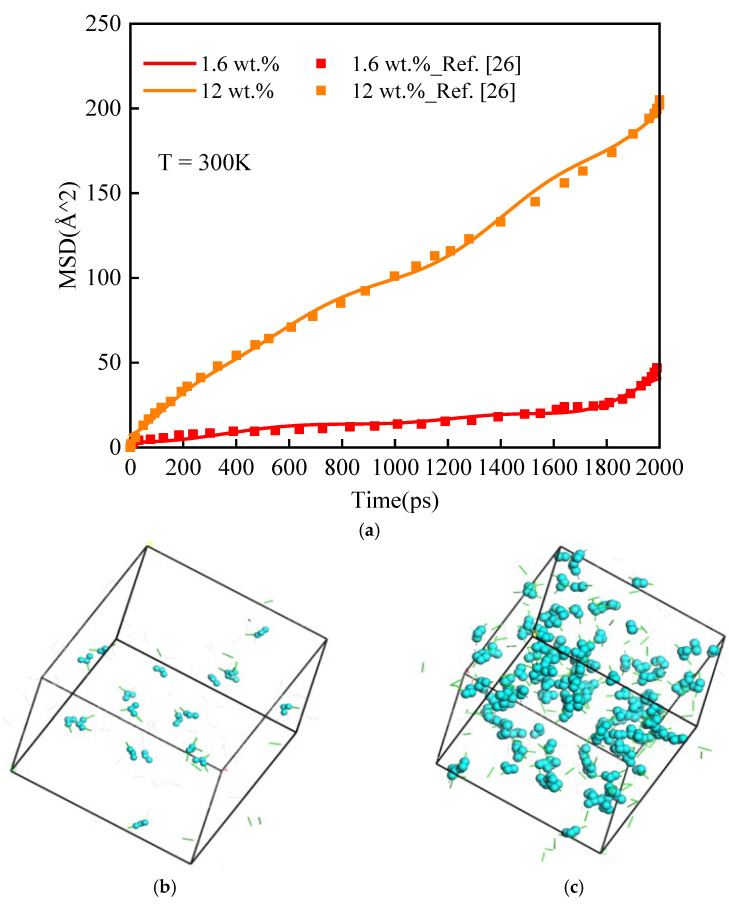
(**a**) Comparison of the MSD. Distribution of water molecules at moisture concentration (**b**) 1.6 wt.% and (**c**) 12 wt.%. The green short lines are water-network hydrogen bonds. The light blue molecules are water molecules.

As shown in Figure 11a, it was found that when the moisture concentration was 12 wt.% or 1.6 wt.%, the mean square displacement in this work was consistent with the reported results by Masoumi [26]. The reason might be that the moisture concentration was lower, and most of the water molecules were bonded with the epoxy polymer by hydrogen bonds [31]. As shown in Figure 11b, when the moisture concentration was 1.6 wt.%, water molecules dispersed among the epoxy polymer. It was consistent with the existence of many NW hydrogen bonds at lower moisture concentrations. With the increase of moisture concentration, the water molecules were mainly free water, which did not form a hydrogen bond with the epoxy polymer [31]. As shown in Figure 11c, when the moisture concentration was 12 wt.%, some water molecules in the polymer gathered together. It was consistent with the existence of many WW hydrogen bonds at higher moisture concentrations. Under these conditions, the MSD of water molecules could hardly be affected by the structures.

### 3.2. Thermal Conductivity

**Figure 12:**
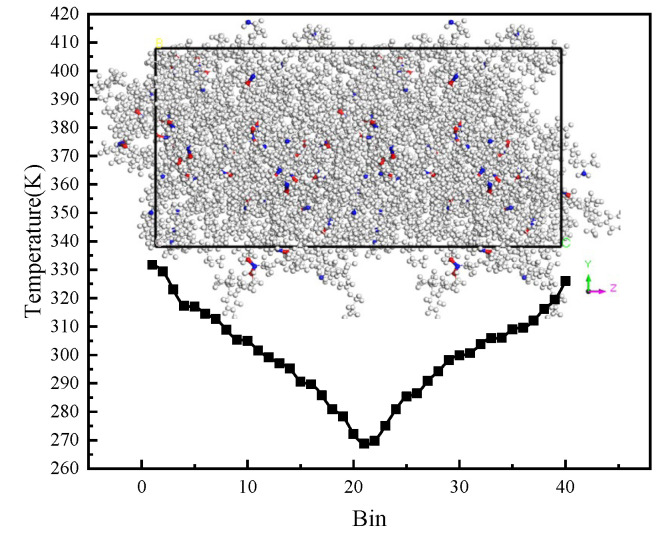
Temperature distribution along the z direction with degree of cross-linking of 85.4%.

**Figure 13:**
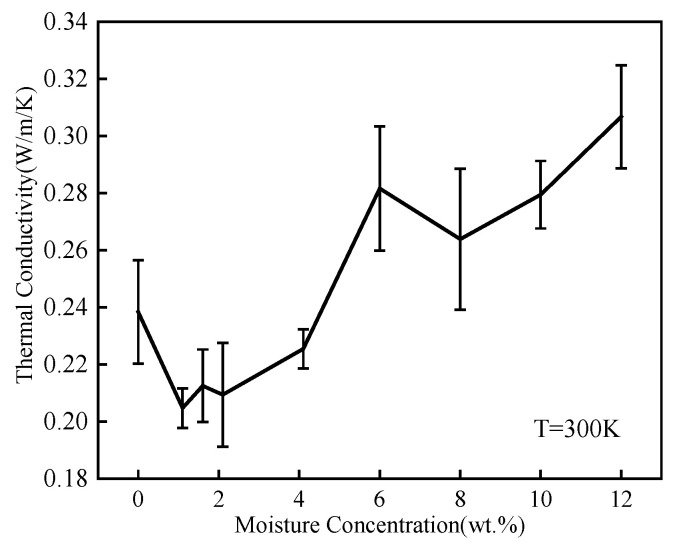
Thermal conductivity for various moisture concentrations.

As a physical quantity of the heat transport of material, the thermal conductivity is an important parameter. The reverse non-equilibrium molecular dynamics (RNEMD) method is usually used to calculate the thermal conductivity [48]. In this work, the RNEMD method was used to calculate the thermal conductivity by MS2017 [35]. Taking into account the size effect of thermal conductivity and the limited calculation ability, the simulated cell was doubled in the z direction (relative to the system size described in Section 2.1). As shown in Figure 12, through the method described in Section 2.3, the temperature of every bin along z direction was obtained. The temperature gradient was obtained by fitting the slope of the temperature curve by the least squares method. The heat flux was calculated through the exchange energy mentioned in Section 2.3). Then, the thermal conductivity was obtained by using the Fourier rule, where the heat flux was proportional to the corresponding temperature gradient. In one-dimension model, it could be expressed as:

$$\kappa \;=\;-\frac{J_{z}}{dT/dz}$$

where, it is assumed that heat flow is only transmitted along the z axis, and *k* is the thermal conductivity. *J_z_* is the heat flux through the z-axis direction. *dT***/***dz* is the temperature gradient in the direction of the z-axis. The minus sign indicates that the heat flows to a lower temperature direction. As shown in Figure 13, the thermal conductivity was calculated three times for each condition, and the average value and standard deviation were obtained. When the moisture concentration was lower than 6 wt.%, the moisture decreased the thermal conductivity. The reason might be that the water molecules caused the epoxy polymer segments to rearrange. As shown in Table 1, the water molecules broke the NN hydrogen bonds, which affected the thermal network chain. The average thermal conductivity generally increased with the increased moisture concentration, indicating the coupling relationship between moisture and thermal properties. There were many gaps between epoxy polymers. The thermal conductivity of the polymers was very low [48]. When water molecules entered the polymers, they either formed hydrogen bonds or filled in the gaps. As the moisture concentration increased, most of the water molecules filled the free volume in the epoxy polymer. This could contribute to the heat to be transferred, and the thermal conductivity would be significantly improved.

### 3.3. Mechanical Response to Uniaxial Tension

**Figure 14:**
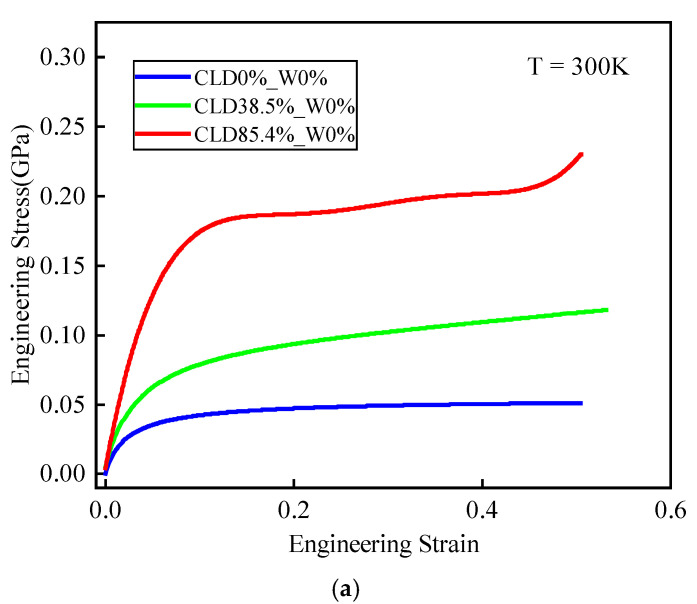

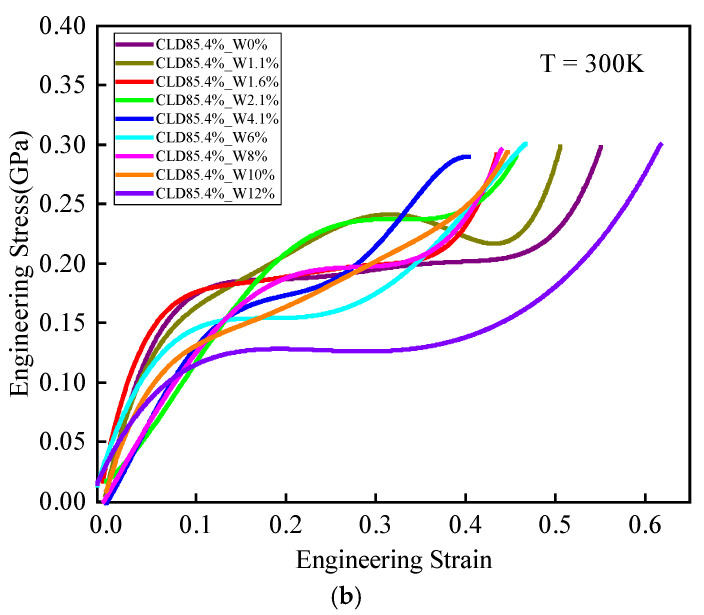
Stress-strain curves for (**a**) various degrees of cross-linking and (**b**) various moisture concentrations. CLD0%_W0% means a system with degree of cross-linking of 0% and moisture concentration of 0 wt.%.

The mechanical properties of the DGEBA/JEFFAMINE^®^-D230 system with different degree of cross-linking and moisture concentrations were studied. The mechanical properties were calculated by using COMPASS and DREIDING force fields and a better result came from the DREIDING force field compared with the experiment. The stress-strain curves calculated by the DREIDING force field are shown in Figure 14. The stress-strain curves did not reveal the fracture of the epoxy polymer, which was partly due to the fact that the force field adopted could not simulate the breakage of chemical bonds. On the other hand, it is due to the fact that the size of the simulated system is very small [49,50,51]. Based on stress-strain curves, Young’s modulus and yield strength of the epoxy polymer were analyzed. Young’s modulus was calculated by linear regression from the stress–strain curve within strains ranging from 0% to 2%. The stress value when the residual deformation reached 0.2% was considered as the conditioned yield strength.

#### 3.3.1. Role of Conversion

**Figure 15:**
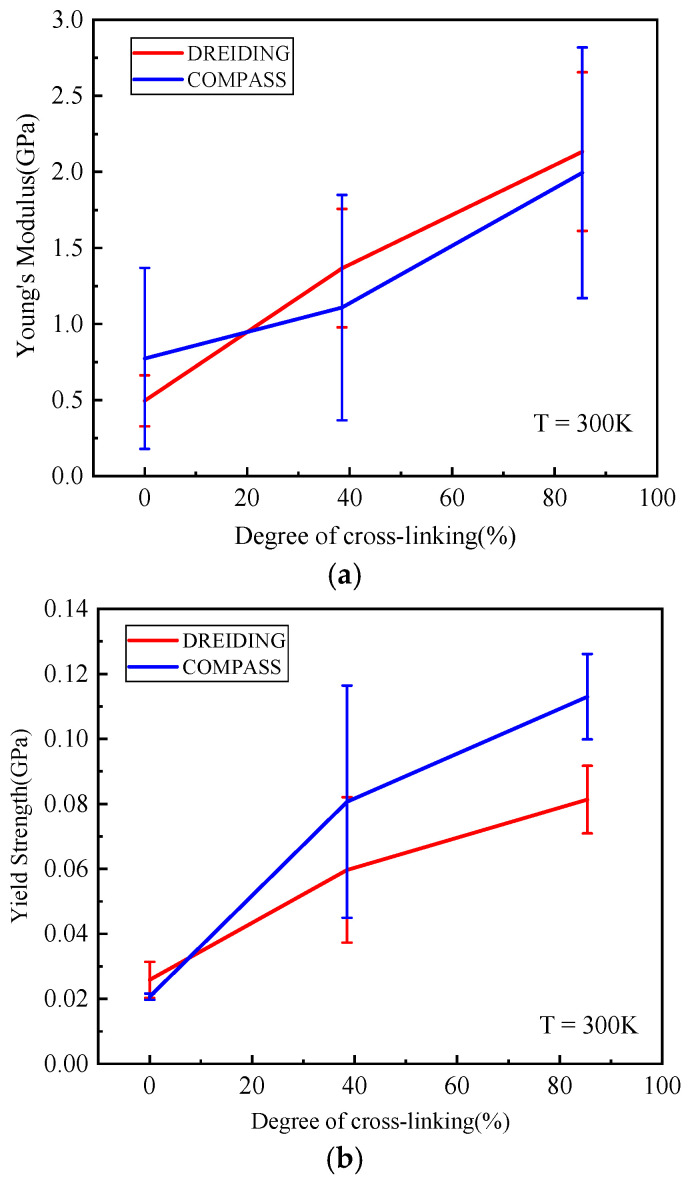
(**a**) Young’s moduli and (**b**) yield strength for various degree of cross-linking.

As shown in Figure 15, the distribution of Young’s moduli and yield strength was wide for each degree of cross-linking in significant overlap. Nonetheless, as the degree of cross-linking increased, the average Young’s moduli and strength of the two force fields both showed an increasing trend. With the increase of degree of cross-linking, the rearrangement ability of the epoxy polymer was weakened under the external load. The higher the degree of cross-linking, the greater the polarity and the stronger the interaction between the chains [34]. As a result, Young’s modulus and the yield stress increased. Sirk et al. [52] used MD simulations to study DGEBA/JEFFAMINE^®^-D230 epoxy at different temperatures and strain rates. Their results showed that the epoxy Young’s modulus was less dependent on strain rate below *T*g. Therefore, as shown in Table 2, Young’s moduli of the epoxy polymer with degree of cross-linking of 85.4% calculated at 300 K was close to the experiment and other simulation results. However, the yield strength was greatly affected by strain rate [34]. As shown in Table 2, the yield strength of the system with a degree of cross-linking of 85.4% was close to other simulation results and experimental values. The results were calculated at 300 K with average rate of 2.4 × 10^8^ s^−1^ in this work and the experiment value was measured at strain rates from 5.7 × 10^−4^ to 8.3 × 10^−3^ s^−1^ [53,54,55].

#### 3.3.2. Role of Moisture Concentration

From the results of Table 2, it was found that the results calculated by DREIDING force field were better. This might be due to two reasons. One is that the hydrogen bond energy was not considered in the COMPASS force field. The second is that it was a more complex force field that contained cross terms [36]. So, when the system had a slight change, it caused the result to be changed a lot. Therefore, only the DREIDING force field was used for this section.

**Figure 16:**
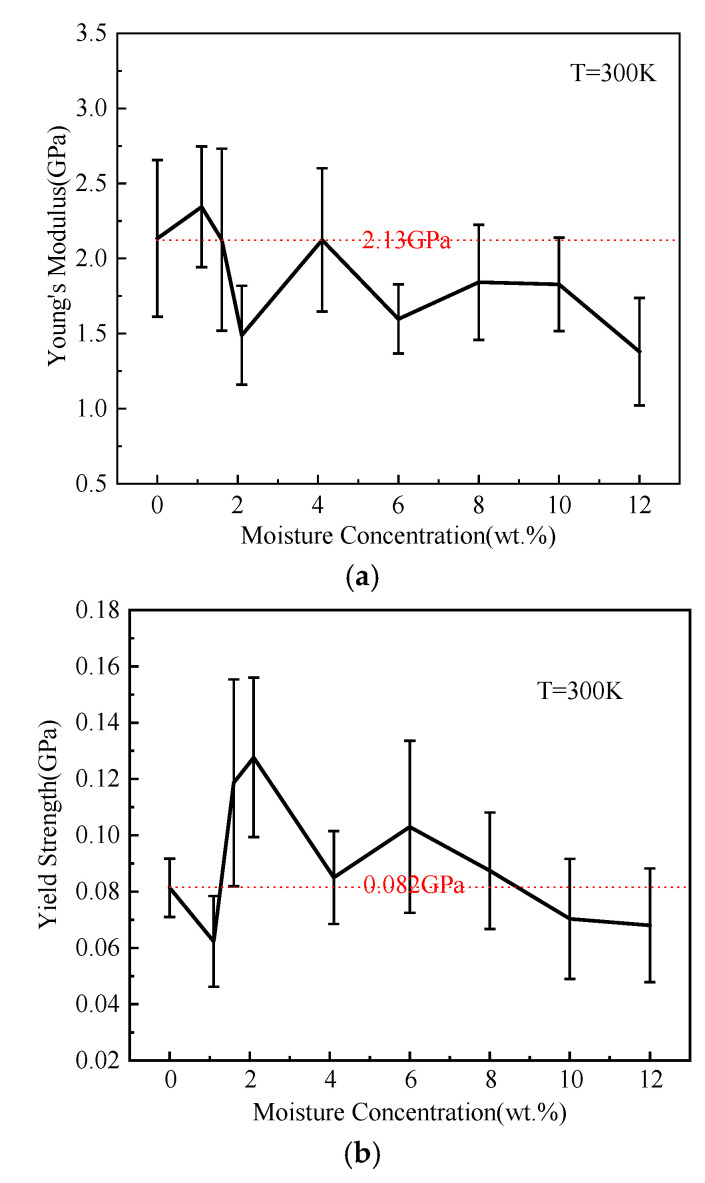
(**a**) Young’s moduli and (**b**) yield strength for various moisture concentrations.

It could be seen from Figure 16a, the general trend was that as the moisture concentration increased, the Young’s modulus decreased. This was due to the plasticization effect of moisture [29,30,58]. The average Young’s moduli range from 1.38 GPa to 2.344 GPa. The reason for the increase of Young’s modulus as the moisture concentration increased was the effect of anti-plasticization [29,30,58]. The moisture in the epoxy polymer affected the motion of the epoxy polymer and the hydrogen bonds. The effect of moisture on Young’s modulus was the result of the competition of various mechanisms. With the increase of moisture concentration, Young’s modulus might change a lot [59,60,61,62,63].

As shown in Figure 16b, when the moisture concentration was smaller than 4.1 wt.%, the yield strength tended to increase with the increase of moisture concentration. After that, the yield strength showed a decreasing trend. The yield strength ranged from 0.062 GPa to 0.128 GPa. The effect of moisture on the yield strength of the epoxy polymer was similar to its effect on the Young’s modulus. The hydrogen bonds between the water molecules and the epoxy polymer resulted in post cross-linking of the system [62]. The improved post cross-linking had greater hindrance on the kinematic ability of the chains. When moisture concentration was below 4.1 wt.%, the NN and WN hydrogen bonds were dominant in the system, resulting in a significant increase in yield strength. With more moisture released into the free volume in the epoxy polymer, it caused the volume to swell. Then, the chains had enough room to move and thereby the yield strength was reduced [62].

## 4. Conclusions

The molecular dynamics method was used to investigate thermo-mechanical properties of cross-linked epoxy resins. The cross-linking between the epoxy resin DGEBA and the curing agent JEFFAMINE^®^-D230 was completed by a multi-step method. The influence of degree of cross-linking and moisture concentration on the thermo-mechanical properties of the epoxy polymer was analyzed.

The interaction between water molecules and epoxy polymer was studied by analyzing the hydrogen bonds, the free volume, and the MSD of water molecules. Most of the hydrogen bonds were related to water molecules as the moisture concentration increased, but the hydrogen bonds between polymers were not reduced a lot. With the increase of moisture concentration, the diffusion coefficient increased. When the moisture concentration reached 12 wt.%, the diffusion coefficient increased exponentially. When the moisture concentration was larger than 12 wt.% or smaller than 1.6 wt.%, the diffusion coefficient was less affected by the epoxy polymer. The decrease rate of free volume slowed down at the moisture concentration of 6 wt.%. The entry of water molecules into the epoxy polymer led to the increase of the thermal conductivity from 0.24 to 0.31 W/m/K. The mechanical properties of the cross-linking system were analyzed by uniaxial tensile simulation, which were calculated by using COMPASS and DREIDING force fields. A better result came from the DREIDING force field compared with the experiment. The increase of degree of cross-linking promoted the increase of mechanical properties. For the system with the largest degree of cross-linking of 85.4%, the Young’s modulus was 2.134 ± 0.522 GPa and the yield strength was 0.081 ± 0.010 GPa. The mechanical properties of the system were affected by both plasticizing and anti-plasticizing when the water molecules entered the polymer. The Young’s modulus and yield strength varied in a large range from 1.38 to 2.344 GPa and from 0.062 to 0.128 GPa, respectively.

There are many interfaces in the electronic package structure, which are the fragile part of the package. In future work, based on the current results, the reliability of the various interfaces will be investigated, such as the interface of the release agent/lead frame, underfill/substrate, and EMC/Cu.

## Figures and Tables

**Table 1:** Number of hydrogen bonds in the system at different moisture concentrations.

Moisture Concentration (wt.%)	WN Hydrogen Bond	NN Hydrogen Bond	WW Hydrogen Bond	Sum
0	0	70	0	70
1.1	24	67	1	92
1.6	35	63	3	101
2.1	47	59	5	111
4.1	70	54	23	147
6	92	52	52	196
8	100	50	78	228
10	118	45	109	272
12	132	43	151	326

**Table 2:** Mechanical properties of DGEBA/JEFFAMINE^®^-D230 with a degree of cross-linking of 85.4%.

Source	Young’s Modulus (GPa)	Yield Strength (GPa)
DREIDING	2.134 ± 0.522	0.081 ± 0.010
COMPASS	1.995 ± 0.824	0.113 ± 0.013
Other simulations	2.087–5.5 GPa [22,34,56]	0.082–0.138 [34]
Experiment	2.47–3.10 [53,54,55,57]	0.057–0.092 [53,54,55]

## Data Availability

The data presented in this study are available on request from the corresponding author.
